# Up-Regulation of CD146 in Schwann Cells Following Peripheral Nerve Injury Modulates Schwann Cell Function in Regeneration

**DOI:** 10.3389/fncel.2021.743532

**Published:** 2021-10-14

**Authors:** Yinying Shen, Jun Zhu, Qianyan Liu, Shiyan Ding, Xinpeng Dun, Jianghong He

**Affiliations:** ^1^Key Laboratory of Neuroregeneration of Jiangsu and Ministry of Education, National Medical Products Administration (NMPA) Key Laboratory for Research and Evaluation of Tissue Engineering Technology Products, Co-innovation Center of Neuroregeneration, Nantong University, Nantong, China; ^2^Department of Thoracic Surgery, Affiliated Hospital of Nantong University, Nantong, China; ^3^Key Laboratory of Acupuncture and Medicine Research of Ministry of Education, School of Medicine and Holistic Integrative Medicine, Nanjing University of Chinese Medicine, Nanjing, China

**Keywords:** CD146, peripheral nerve injury, up-regulation, Schwann cells, blood vessels, regeneration

## Abstract

CD146 is cell adhesion molecule and is implicated in a variety of physiological and pathological processes. However, the involvement of CD146 in peripheral nerve regeneration has not been studied yet. Here, we examine the spatial and temporal expression pattern of CD146 in injured mouse sciatic nerve via high-throughput data analysis, RT-PCR and immunostaining. By microarray data analysis and RT-PCR validation, we show that CD146 mRNA is significantly up-regulated in the nerve bridge and in the distal nerve stump following mouse sciatic nerve transection injury. By single cell sequencing data analysis and immunostaining, we demonstrate that CD146 is up-regulated in Schwann cells and cells associated with blood vessels following mouse peripheral nerve injury. Bioinformatic analysis revealed that CD146 not only has a key role in promoting of blood vessel regeneration but also regulates cell migration. The biological function of CD146 in Schwann cells was further investigated by knockdown of CD146 in rat primary Schwann cells. Functional assessments showed that knockdown of CD146 decreases viability and proliferation of Schwann cells but increases Schwann cell migration. Collectively, our findings imply that CD146 could be a key cell adhesion molecule that is up-regulated in injured peripheral nerves to regulate peripheral nerve regeneration.

## Introduction

Successful peripheral regeneration requires the co-ordination of injured neurons and multiple cell types in the distal nerve stump (Chen et al., 2007; Zigmond and Echevarria, 2019; Min et al., 2020). Damaged neurons shift from action potential transduction mode to a growth mode while Schwann cells, resident macrophages and endoneurial fibroblasts in the distal nerve stump undergo remarkable phenotypic changes in order to create a growth permissive environment for axon regeneration (Weng et al., 2018; Jessen and Arthur-Farraj, 2019; Zigmond and Echevarria, 2019; Toma et al., 2020). Both myelinating and non-myelinating Schwann cells from injured site to the entire distal nerve stump dedifferentiate and proliferate to become mesenchymal-like repair cells and support axon elongation (Jessen and Mirsky, 2016). In the case of nerve transection injury, Schwann cells from both nerve ends migrate into the nerve gap and build Schwann cell cord to guide regenerating axons across the nerve gap (Parrinello et al., 2010; Clements et al., 2017; Chen et al., 2019; Min et al., 2020). The high plasticity of Schwann cells is one of the key reasons that the peripheral nervous system is superior over the central nervous system in regeneration following damage (Vargas and Barres, 2007; Chen et al., 2007; Jessen and Mirsky, 2016). Many neurotrophins, growth factors and cell adhesion molecules have been discovered to be differentially expressed in Schwann cells after peripheral nerve injury, and they have been demonstrated as important modulators of Schwann cell phenotype and peripheral nerve regeneration (Madduri and Gander, 2012; Jessen and Mirsky, 2016; Zhang et al., 2019). For instance, vascular endothelial growth factor stimulates the survival and proliferation of Schwann cells (Sondell et al., 1999). Nerve growth factor (NGF) promotes sensory axon regeneration while brain-derived neurotrophic factor (BDNF) and glial cell line-derived neurotrophic factor (GDNF) promote motor axon regeneration (Hoyng et al., 2014; Min et al., 2020). In contrast, neuregulin and betacellulin enhance Schwann cell proliferation, migration and remeylination (Li et al., 2015; Yi et al., 2016; Vallieres et al., 2017; Wang et al., 2021).

CD146, a cell surface protein encoded by MCAM (melanoma cell adhesion molecule), not only acts as a cell adhesion molecule but also is a signaling receptor of many extracellular matrix-related proteins and growth factors (Wang and Yan, 2013; Wang Z. et al., 2020). CD146 mediates multiple activities of various cell types including endothelial cells, epithelial cells, macrophages and T cells, and participates in miscellaneous biological processes such as development, angiogenesis and immune responses (Chen et al., 2017; Luo et al., 2017; Leroyer et al., 2019). The expression of CD146 is low in adult tissues but is highly expressed under various pathological conditions, especially carcinoma (Shih, 1999; Luo et al., 2012; Yawata et al., 2019). Previously, we performed sequencing analysis and studied the time course of gene expression profile in rat sciatic nerves (SRP113121) (Yi et al., 2015). Sequencing data analysis identified CD146 as one of differentially expressed molecules following peripheral nerve injury. To understand the expression pattern and a possible function of CD146 in peripheral nerve regeneration, in this study, we examined the dynamic changes of CD146 expression in injured mouse sciatic nerve, and investigated cell type specific distributions of CD146. We identified that CD146 is up-regulated in Schwann cells and cells associated with blood vessels in the nerve bridge and the distal nerve stump. Using cultured rat primary Schwann cells with siRNA transfection against CD146, we further demonstrated the regulatory role of CD146 in Schwann cell viability, proliferation and migration.

## Materials and Methods

### Computational Analysis of Microarray and Single Cell Sequencing Data Sets

Microarray data of mouse distal sciatic nerves at 0, 3, 7, and 14 days after sciatic nerve crush injury were downloaded from NCBI GEO database GSE74087 (Pan et al., 2017) and GSE22291 (Barrette et al., 2010). Normalized data was applied to determine the fold changes of CD146 in the injured mouse sciatic nerves. Single cell sequencing data for intact mouse sciatic nerve and for the distal nerve at day 3 after sciatic nerve transection injury were downloaded from GSE147285 (Toma et al., 2020). Single cell sequencing data for mouse distal nerve at day 9 after sciatic nerve transection injury were downloaded from GSE120678 (Carr et al., 2019). Data were analyzed using Seurat v.3.2.1^1^ and sctransform v.0.3 R packages v.4.0.2 as previously described (Chen et al., 2021). CD146 dotplot, tSNE and violinplot were created using Seurat specific function.

### Animal Surgery

Animal experiments were ethical approved by the Administration Committee of Experimental Animals, China. Experimental procedures were carried out according to Institutional Animal Care Guidelines of Nantong University. Adult (2 month old) proteolipid protein (PLP)-GFP mice (Mallon et al., 2002) and C57BL/6 mice were subjected to sciatic nerve transection injury as previously described (Dun and Parkinson, 2018). Briefly, after isoflurane anesthetization and sciatic nerve exposure, sciatic nerve was transected at approximately 0.5 cm proximal to the nerve trifurcation site. Muscles were closed with sutures and skin was closed with Autoclips. Mice were given post-operative analgesia and sacrificed by CO_2_ at 7 days after surgery. In total, 12 C57BL/6 mice and 3 PLP-GFP mice were used in this study.

### Immunostaining

Sciatic nerves were collected and fixed with 4% paraformaldehyde overnight at 4°C. Fixed nerves were washed with PBS and dehydrated with 30% sucrose, and then embedded in OCT medium. Embedded nerves were sectioned on a cryostat at a thickness of 12 μm. Nerve sections were permeabilized with 0.25% Triton X-100 containing 1% BSA in PBS, blocked with blocking buffer (0.05% Triton X-100 containing3% BSA in PBS), and incubated with primary antibodies and species-specific secondary antibodies plus Hoechst 33342 nuclear dye diluted in blocking buffer. Primary antibodies neurofilament heavy chain (ab4680) and CD146 (ab75769) were purchased from Abcam (Cambridge, United Kingdom), CD31 (#5550274) was purchased from BD Pharmingen, NeuN was purchased from Merck Millipore (ABN91). Alexa Fluor 488 or 568 dye-conjugated donkey anti-rabbit and goat anti-chicken secondary antibodies and Hoechst 33342 nuclear dye (H3570) were purchased from Thermo Scientific (Basingstoke, United Kingdom). Stained nerve sections were mounted with Citifluor (Agar Scientific, R1320) and images were taken using a Leica SPE confocal microscope.

### Bioinformatic Analysis

Functional pathways of CD146 were revealed using Ingenuity pathway analysis (IPA; Ingenuity Systems Inc., Redwood City, CA, United States) according to the build-in Ingenuity Pathways Knowledge Base (Yi et al., 2015). Interaction network of CD146, interacted factors and Gene Ontology (GO) biological processes were obtained using the ClueGo plug-in in the Cytoscape software.

### Schwann Cell Culture and siRNA Transfection

Schwann cells were isolated from neonatal rat sciatic nerves, purified with anti-Thy1.1 (1:1,000, M7898, Sigma, St. Louis, MO, United States) and rabbit complement (Invitrogen, Carlsbad, CA, United States). Purified Schwann cells were cultured in DMEM (10-013-CVR, Corning, NY, United States) supplemented with 10% FBS (10099141c, Gibco, Grand Island, NY, United States). Cultured cells were transfected with CD146 siRNA (sequence: TAGTCAAGGAGGACAAAGA) or control siRNA (random sequence; RiboBio, Guangzhou, Guangdong, China) using Lipofectamine RNAiMAX transfection reagent (Invitrogen).

### RT-PCR

Total mRNA was extracted from dissected left side sciatic nerve (control), nerve bridge and the distal nerve using a miRNeasy Mini Kit (Qiagen, Hilden, Germany) and first stand cDNA was synthesized with M-MLV reverse transcriptase (Promega, Southampton, United Kingdom) using random hexamer primers (Promega). Total mRNA was isolated from cultured cells using RNA-Quick Purification Kit (Yishan Biotechnology Co., Shanghai, China), reversely transcribed using HiScript III RT SuperMix for qPCR (+ gDNA wiper) (Vazyme, Nanjing, Jiangsu, China), and applied to a StepOne Real-time PCR System (Applied Biosystems, Foster City, CA, United States) with ChamQ SYBR qPCR Master Mix (Vazyme, Nanjing, Jiangsu, China). The sequences of primer pairs were as follows: CD146 (forward: 5′-AGGACCTTGAGTTTGAGTGG-3′; reverse: 5′-CAGTGGTTTGGCTGGAGT-3′), P0 (forward: 5′-CGTGATCG GTGGCATCCTC-3′; reverse: 5′- GGCATACAGCACTGGCG TCT-3′), p75 (forward: 5′-CTGCTGATTCTAGGGATGTCCT-3′; reverse: 5′-ATGTAACACTGTCCAGGCAGG-3′), GAPDH (forward: 5′-ACAGCAACAGGGTGGTGGAC-3′; reverse: 5′-TTTGAGGGTGCAGCGAACTT-3′), and 18 s (forward: 5′-GAGAAACGGCTACCACATCC-3′; reverse: 5′-GGACACT CAGCTAAGAGCATCG-3′). The ΔΔCt method was applied to quantify the relative abundances of CD146, P0, and p75 with GAPDH or 18 s as the reference gene.

### Cell Viability Assay

Schwann cells transfected with CD146 siRNA or control siRNA for 36 h were seeded on 96-well plates at a density of 2 × 10^5^ cells/ml and exposed to 10 μl of CCK8 using Cell Counting Kit-8 (Beyotime, Shanghai, China). After 2 h culture, cell viability was measured spectrophotometrically at 450 nm using a Synergy^TM^ 2 Multi-Mode Microplate Reader (BioTek, Burlington, VT, United States). Experiment was conducted four times using independent cultures, each tested induplicate.

### Cell Proliferation Assay

Transfected Schwann cells were seeded onto 96-well plates at a density of 2 × 10^5^ cells/ml and exposed to 50 μM EdU for 14 h using Cell-Light EdU DNA Cell Proliferation Kit (Ribibio). After additional 24 h, cells were fixed with 4% paraformaldehyde and stained with EdU and Hoechst 33342. Images were taken under Leica Model DMi8 (Leica Microsystems CMS GmbH, Bensheim, Germany). Experiment was conducted three times using independent cultures, each tested induplicate.

### Cell Migration Assay

A 6.5 mm Transwell chamber with 8 μm pores (Costar, Cambridge, MA, United States) was applied to determine cell migration ability. The upper chamber was filled with transfected Schwann cells suspended in 100 μl DMEM at a density of 4 × 10^5^ cells/ml. The bottom chamber was filled with 500 μl FBS containing cell culture medium. After 24 h culture, non-migrated Schwann cells on the upper surface of each membrane were cleaned with a cotton swab while migrated Schwann cells adhering to the bottom surface were stained with 0.1% crystal violet. Images were taken under Leica Model DMI3000B (Leica Microsystems). Experiment was conducted three times using independent cultures, each tested in triplicate.

A mold chamber with a 1 mm wide insert placed in the middle of the chamber was also applied. Transfected Schwann cells were seeded onto the two rectangular culture spaces in the mold chamber at a density of 2 × 10^5^ cells/ml. Schwann cells were cultured for additional 9 h after cell confluence and insert removal. Remaining blank space was measured using Image-Pro Plus (Media Cybernetics, Silver Spring, MD, United States). Images were taken under Leica Model DMI3000B. Experiment was conducted three times using independent cultures, each tested induplicate or triplicate.

### Statistical Analysis

Summarized data were presented as means ± SEM. Statistical significance was analyzed using student’s *t*-test or one-way ANOVA followed by Dunnett’s multiple comparisons test. Graphs and statistical tests were performed with GraphPad Prism 6.0 (GraphPad Software, Inc., La Jolla, CA, United States).

## Results

### Enhanced Expression of CD146 After Peripheral Nerve Injury

**FIGURE 1:**
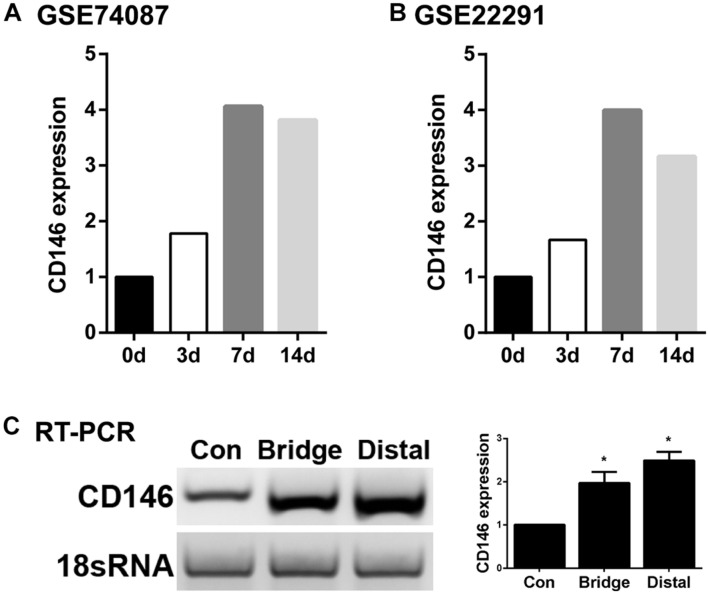
Up-regulation of CD146 in injured mouse peripheral nerves. **(A,B)** CD146 fold changes in the distal nerve at 3, 7, and 14 days after mouse sciatic nerve injury, data was analyzed from microarray datasets GSE74087 **(A)** and GSE22291 **(B)**. **(C)** RT-PCR detection of CD146 expressions in intact mouse sciatic nerve (con), and in the nerve bridge and the distal nerve at day 7 after mouse sciatic nerve transection injury. Biological replicates *n* = 3, *p-value < 0.05.

To examine CD146 expression in the distal nerve stump following mouse sciatic nerve injury, we first analyzed two published microarray data sets, GSE74087 (Pan et al., 2017) and GSE22291 (Barrette et al., 2010). Both data sets have studied gene expression profile in the distal nerve at 0, 3, 7 and 14 days following mouse sciatic nerve crush injury. Consistently, both data set revealed that CD146 was significantly up-regulated in the distal nerve at 3, 7 and 14 days following mouse sciatic nerve crush injury (Figures 1A,B). In microarray dataset GSE74087 (Pan et al., 2017), CD146 showed approximately 1.8 fold up-regulation at day 3 after injury as compared with its expression in uninjured mouse sciatic nerve (0 day). CD146 expression increased to an even higher level at day 7 and day 14 (Figure 1A). Similarly, data set GSE22291 (Barrette et al., 2010) showed approximately 1.7 fold up-regulation at day 3 after injury as compared with its expression in uninjured mouse sciatic nerve (0 day). CD146 up-regulation peaks at day 7 post-injury with both data set showing around fourfold up-regulation (Figures 1A,B). At day 14 post-injury, CD146 is slightly down-regulated compared with its up-regulation at day 7 post-injury but remain more than threefold up-regulation (Figures 1A,B). Because both data sets show the highest fold change of CD146 at day 7 post-injury, we performed RT-PCR study and validated our analysis using nerve bridge tissue and the distal nerve samples at day 7 after mouse sciatic nerve transection injury with left site uninjured sciatic nerve acting as control samples. In line with these observations by microarray data analysis, our RT-PCR results showed significant CD146 up-regulation in the nerve bridge and in the distal nerve stump at day 7 post-injury (Figure 1C). Our RT-PCR results also showed that CD146 is expressed in intact mouse sciatic nerves and it is further up-regulated in response to injury.

**FIGURE 2:**
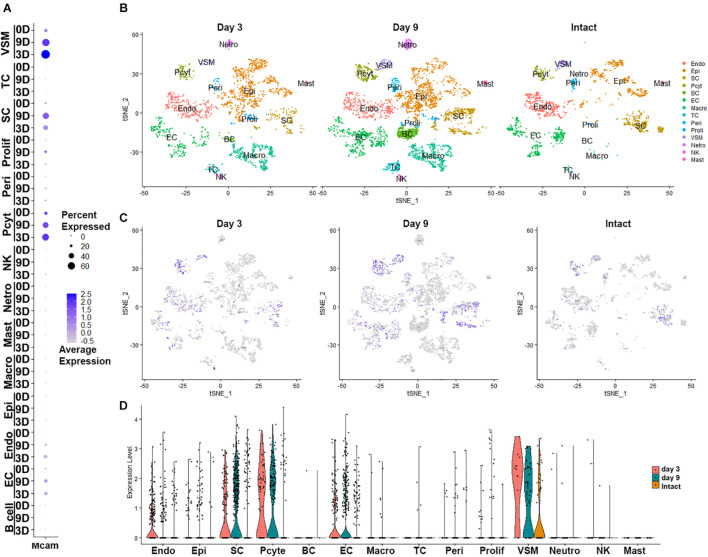
Cell type specific expression of CD146 in intact and injured mouse sciatic nerve. **(A)** Dot plot showing CD146 up-regulation Schwann cells, VSM cells, perictyes and endothelial cells. **(B–D)** tSNE and violin plot showing Cell type specific expression of CD146 in intact and injured mouse sciatic nerve. Single cell sequencing data sets were analyzed from GSE147285 and GSE120678. EC, Endothelial cells; VSM, Vascular smooth muscle cells; Pcyt, Pericytes; Endo, Endoneurial fibroblasts; Peri, Perineurial fibroblasts; Epi, Epineurial fibroblasts; SC, Schwann cells; Macro, Macrophages; Neutro, Neutrophils; Mast, Mast cells; TC, T cells; NK, NK cells.

Next, we examined cell type specific expression of CD146 in both intact and injured mouse sciatic nerves with analyzed single cell RNA sequencing data GSE147285, which studied cell types and gene expression in intact mouse sciatic nerve and in the distal sciatic nerve at day 3 after sciatic nerve transection injury (Toma et al., 2020; Chen et al., 2021), and GSE120678 which studied cell types and gene expression in the distal sciatic nerves at day 9 after sciatic nerve transection injury (Carr et al., 2019; Chen et al., 2021). Single cell transcriptomics analysis demonstrated that CD146 is expressed in non-myelinating Schwann cells, vasculature-associated smooth muscle (VSM) cells and pericytes of intact mouse sciatic nerve (Figure 2 and Table 1). Following injury, CD146 is strongly up-regulated in Schwann cells, VSM cells and pericytes of the distal nerve. It is also up-regulated in endoneurial fibroblasts and endothelial cells (Figure 2 and Table 1). Thus, the single cell transcriptomics analysis demonstrated that CD146 is highly expressed in Schwann cells of the distal nerve and cells associated with blood vessels in the distal nerve stump.

**TABLE 1:** Expression levels of CD146 in cells of intact and injured mouse sciatic nerve.

	Schwann cells		VSM		Pericytes		Endothelial cells		Endoneurial cells	
	%	AE	%	AE	%	AE	%	AE	%	AE
Intact	13.65	1.02	25.88	1.16	23.47	1.61	13.53	0.45	6.22	0.23
Day 3	36.96	1.13	71.43	2.23	54.05	1.75	26.32	0.83	31.49	0.6
Day 9	49.66	1.55	57.14	1.74	48	1.64	25.86	1.03	16.54	0.47

	**Perineurial cells**		**Epineurial cells**		**neutrophils**		**NK cells**		**T cells**	
	**%**	**AE**	**%**	**AE**	**%**	**AE**	**%**	**AE**	**%**	**AE**

Intact	9.86	0.44								
Day 3	14.81	0.45	2.69	0.09	4.55	0.45	7.41	0.87		
Day 9	11.49	0.47	5.61	0.23			2.44	0.11	2.45	0.17

*VSM, Vasculature-associated smooth muscle cells; AE, average expression.*

### Expression of CD146 in Cells of Injured Mouse Sciatic Nerve

**FIGURE 3:**
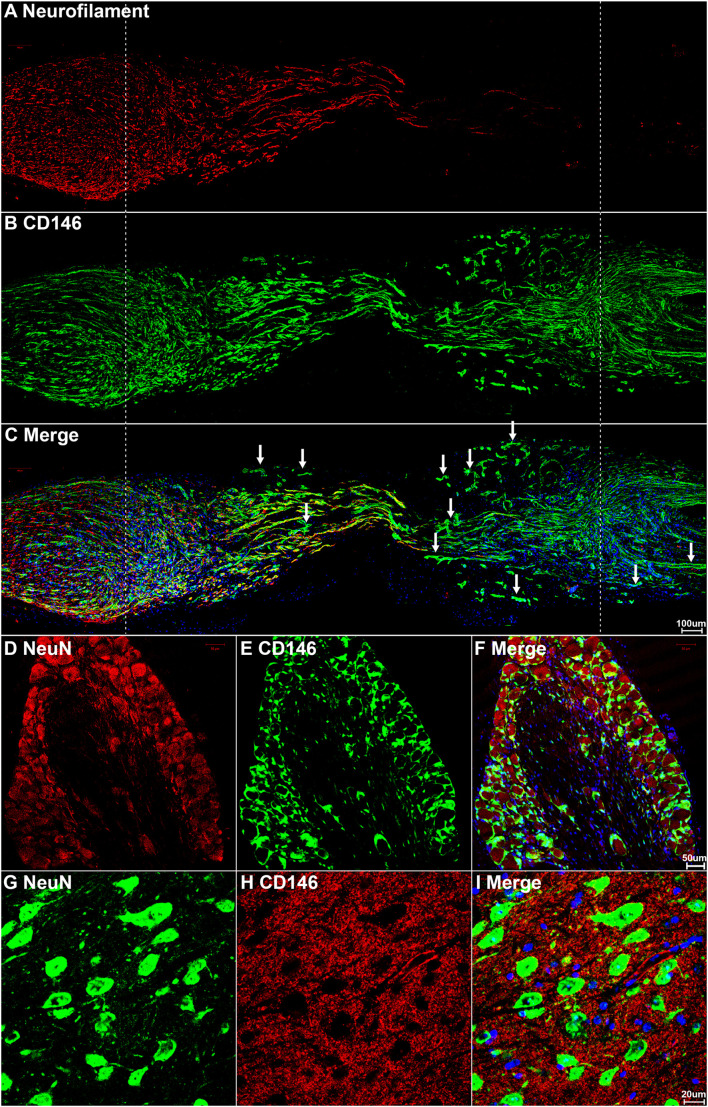
CD146 expression in mouse peripheral nervous system following sciatic nerve transection injury. **(A–C)** Neurofilament and CD146 double staining on day 7 sciatic nerve longitudinal sections showing that CD146 is not expressed in regenerating axons. Scale bar in **(C)** 200 μm. Proximal nerve is located on the left and distal nerve is located on the right. Nerve bridge is located between two dished lines. **(D–F)** NeuN and CD146 double staining showing that CD146 is not expressed in sensory neurons of DRG. Scale bar in **(F)** 50 μm. **(G–I)** NeuN and CD146 double staining showing that CD146 is not expressed in motor neurons of spinal cord. Scale bar in **(I)** 20 μm. Biological replicates *n* = 3.

The expression of CD146 in cells of the injured mouse sciatic nerve was further investigated by immunohistochemistry after sciatic nerve transection injury. First, we double stained CD146 with an axon marker, neurofilament heavy chain, to reveal CD146 expression in transected mouse sciatic nerve at day 7 post-injury. The staining results revealed that CD146 doesn’t co-localize with axons in the proximal nerve stump, indicating that regenerating axons don’t express CD146 (Figure 3C). To confirm that regenerating axons don’t express CD146, we double stained CD146 with a neuronal marker NeuN on sections of both spinal cord and dorsal root ganglion (DRG) after sciatic nerve transection injury. The staining confirmed that cell bodies of both motor neurons in the ventral horn of spinal cord (Figures 3G–I) and sensory neurons in DRG don’t express CD146 (Figures 3D–F). At day 7 post-injury, regenerating axons have extended into the middle of the nerve bridge (Figure 3A). Interestingly, CD146staining signal partially co-localizes with regenerating axons in the nerve bridge (Figures 3A–C) although neurons do not express CD146. Regenerating axons in the nerve bridge are known to attach migrating Schwann cells for elongation (Chen et al., 2019; Min et al., 2020), indicating that CD146 could be expressed in migrating Schwann cells inside the nerve bridge. This staining also showed that CD146 is highly expressed in the distal nerve stump (Figure 3B).

**FIGURE 4:**
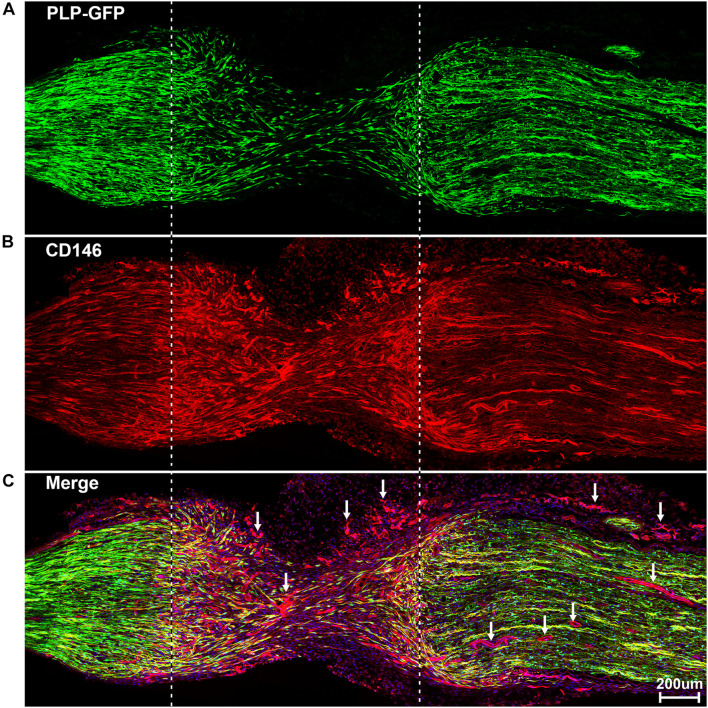
Staining of CD146 on longitudinal sciatic nerve sections of PLP-GFP mice at day 7 after sciatic nerve transection injury showing CD146 expression in Schwann cells of the nerve bridge and the distal nerve stump. **(A)** PLP-GFP signal in Schwann cells. **(B)** CD146 staining in red. **(C)** Merged signals including Hoechst staining. Proximal nerve is located on the left and distal nerve is located on the right. Nerve bridge is located between two dished lines. Arrows in **(C)** indicated CD146 expression in blood vessels. Scale bar in **(C)** 200 μm. Biological replicates *n* = 3.

**FIGURE 5:**
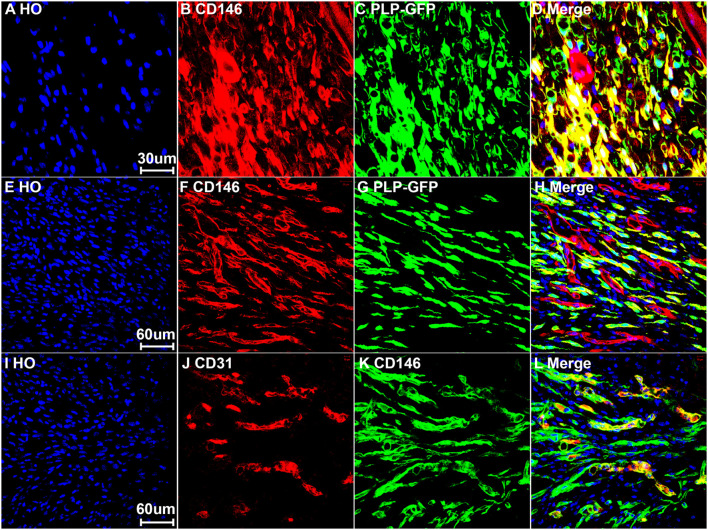
Expression of CD146 in Schwann cells and cells associated with blood vessels of mouse sciatic nerve at day 7 post-injury. **(A–D)** Staining CD146 on distal nerve transverse sections. **(E–H)** CD146 on longitudinal nerve bridge sections from PLP-GFP mice at day 7 day after sciatic nerve transection injury showing migrating Schwann cells in the nerve bridge express CD146. Scale bar in **(E)** 60 μm. **(I–L)** Double staining CD146 with CD31 on longitudinal nerve bridge sections from C57BL/6 mice at day 7 after transection injury showing cells associated with blood vessels express CD146. Scale bar in **(I)** 60 μm. Biological replicates *n* = 3.

To reveal CD146 up-regulation in Schwann cells, we stained CD146 on sciatic nerve sections at day 7 post-injury from PLP-GFP mice which Schwann cells are labeled with GFP (Mallon et al., 2002; Dun et al., 2019). As revealed by the single cell sequencing data analysis that CD146 is up-regulated in Schwann cells, strong CD146 immunostaining signals not only could be observed in GFP positive Schwann cells of the distal nerve stump (Figures 4, 5A–D) but also could be observed in GFP positive Schwann cells inside the nerve bridge (Figures 4, 5E–H). The staining results confirmed that Schwann cells in the distal nerve stump and migrating Schwann cells inside the nerve bridge express high level of CD146.

Above neurofilament double staining as well as CD146 staining in the PLP-GFP mice also showed that CD146 is strongly expressed in another population cells in the nerve bridge and in the distal nerve stump (indicated by arrows in Figures 3C, 4C). Morphologically, these cells are associated with blood vessels. Our single cell sequencing data analysis showed that CD146 is highly up-regulated in VSM cells, pericytes and endothelial cells (Figure 2 and Table 1). Due to the limitation of available antibodies for double immunostaining, we therefore double stained CD146 with an endothelial cell marker CD31 to reveal CD146 expression in blood vessels. In consistent with our single cell sequencing data analysis, CD146 is highly expressed in cells associated with blood vessels (Figures 5I–L). Thus, our immunostaining results confirmed our single cell sequencing data analysis that, following peripheral nerve injury, CD146 is highly expressed in Schwann cells and cells associated with blood vessels.

### Identifying Interaction Network of CD146

**FIGURE 6:**
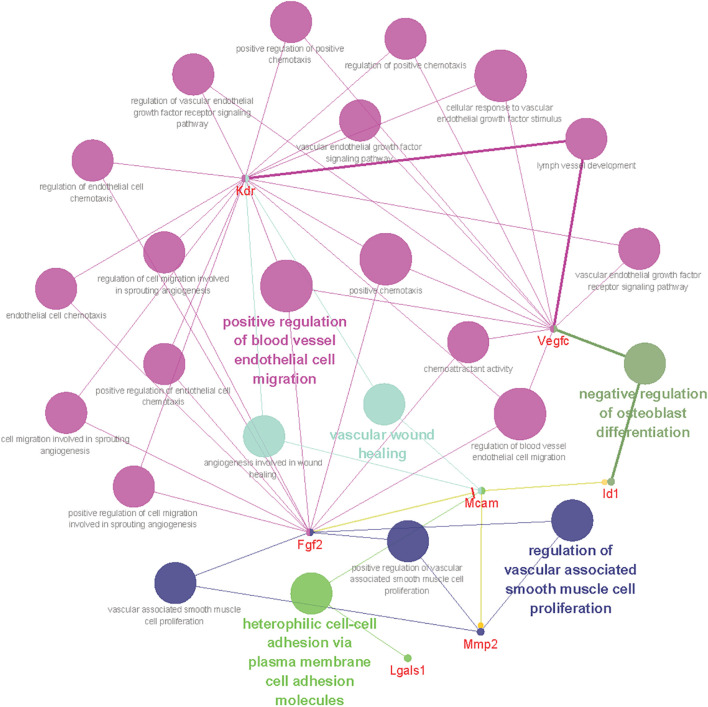
Bioinformatic analysis showing CD146 functional network during peripheral nerve regeneration. Different colors of network nodes, respectively, represent different GO (Gene Ontology), with rose red representing GO:0043536 positive regulation of blood vessel endothelial cell migration, bright green representing GO:0007157 heterophilic cell-cell adhesion via plasma membrane cell adhesion molecules, dark green representing GO:0045668 negative regulation of osteoblast differentiation, light green representing GO:0061042 vascular wound healing, and blue representing GO: 1904705 regulation of vascular associated smooth muscle cell proliferation. The size of the nodes represents *p*-value.

To gain insight into the biological function of CD146 up-regulation in injured peripheral nerves, the functional networks were investigated according to our sequencing data of injured rat sciatic nerves (Yi et al., 2015). Significant up-regulated genes were categorized based on the biological functions of CD146 using Gene Ontology (Figure 6). The functional network analysis revealed two important biological functions for CD146 in peripheral nerve regeneration. One biological function for CD146 is to promote angiogenesis (Figure 6). The up-regulation of CD146 in cells associated with regenerating blood vessels in the nerve bridge and the network analysis results indicate that CD146 could take part blood vessel regeneration in the nerve bridge by interacting with VEGF and FGF2 signaling pathways (Figure 6). In line with this finding, CD146 has a well characterized function in promoting angiogenesis and vascular development (St Croix, 2015; Tu et al., 2015). The other biological function for CD146 is to regulate cell migration (Figure 6). In view of the high abundance of CD146 in Schwann cells after peripheral nerve injury and the essential roles of migrating Schwann cells in the nerve bridge to direct axon regeneration, the biological functions of CD146 on Schwann cells were further examined in the following studies.

### Biological Functions of CD146 in Schwann Cells

**FIGURE 7:**
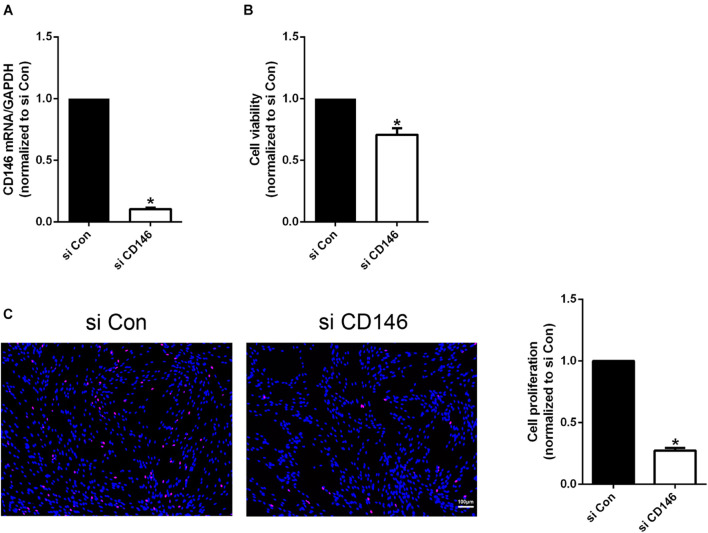
Effects of CD146 on Schwann cell viability, proliferation and differentiation. **(A)** Efficiency of CD146 siRNA on CD146 expression in culture rat primary Schwann cells. Biological replicates *n* = 3. **(B)** Reduced Schwann cell viability after CD146 siRNA transfection. Biological replicates *n* = 4. **(C)** Proliferation rate of Schwann cells after CD146 siRNA transfection. Scale bar in C 100 μm. Biological replicates *n* = 3. The statistical differences were analyzed by student’s *t*-test for comparisons between two groups or one-way analysis of variance (ANOVA) followed by Dunnett’s multiple comparisons test for comparisons between multiple groups using GraphPad Prism 6.0 software (GraphPad Software, La Jolla, CA). *p-value < 0.05.

CD146 is highly expressed in Schwann cells of the injured peripheral nerves. Cultured Schwann cells are known to express similar gene as Schwann cells in the distal nerve stump (Jessen and Mirsky, 2016). Our qPCR result also confirmed that cultured rat primary Schwann cells express high level of CD146 (Figure 7A). We therefore took an *in vitro* approach to investigate CD146 function by knockdown CD146 with CD146 siRNA in cultured rat primary Schwann cells. The efficiency of CD146 knockdown was validated by qPCR to compare CD146 mRNA levels between control siRNA and CD146 siRNA transfected Schwann cells. qPCR results show that CD146 was effectively knocked down in Schwann cells following CD146 siRNA transfection (Figure 7A). After the validation of effective CD146 knockdown in Schwann cells, the function of CD146 on Schwann cell viability, proliferation and migration was investigated. CCK8 experiments showed that Schwann cells with attenuated CD146 expression exhibited relatively lower cell viability (Figure 7B). EdU proliferation assay demonstrated that after transfection with CD146 siRNA, the number of EdU-labeled proliferating cells and the ratio of proliferating cells to total cells were robustly reduced (Figure 7C). These findings indicate that knockdown of CD146 impairs the viability and proliferation of Schwann cells.

**FIGURE 8:**
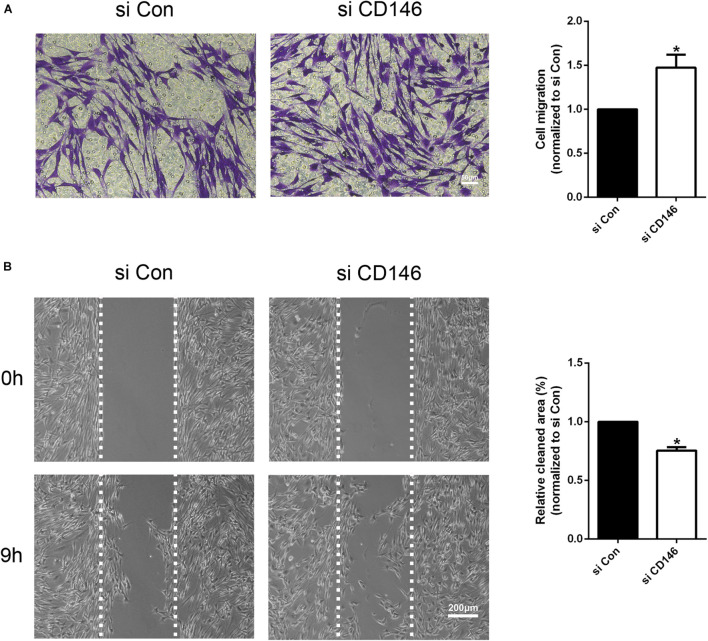
Effects of CD146 on Schwann cell migration. **(A)** The Transwell-crossing migration ability of Schwann cells after CD146 siRNA transfection. Scale bar indicates 50 μm. Biological replicates *n* = 3. **(B)** The wound healing ability of Schwann cells after CD146 siRNA transfection. Scale bar indicates 200 μm. Biological replicates *n* = 3. The statistical differences were analyzed by student’s *t*-test for comparisons between two groups or one-way analysis of variance (ANOVA) followed by Dunnett’s multiple comparisons test for comparisons between multiple groups using GraphPad Prism 6.0 software (GraphPad Software, La Jolla, CA). *p-value < 0.05.

The high level of CD146 expression in migratory Schwann cells in the nerve bridge indicates that CD146 could regulate Schwann cell migration. Finally, we examined the effect of CD146 on Schwann cell migration by Transwell migration assay and wound healing assay. In control siRNA transfected Schwann cells after 24 h culture, a large amount of Schwann cells could be detected that have migrated across the upper surface (Figure 8A). In contrast, in CD146 siRNA transfected Schwann cells, significant more Schwann cells could be detected in the bottom surface of Transwell, suggesting that CD146 knockdown stimulates Schwann cell migration (Figure 8A). In line with the finding of Transwell migration assay, wound healing assay also showed enhanced Schwann cell migration following CD146 knockdown (Figure 8B).

## Discussion

CD146 (cluster of differentiation 146) is a cell adhesion molecule and it belongs to the immunoglobulin superfamily (Wang and Yan, 2013). It was initially identified as a tumor marker for melanoma with strong expression on metastatic lesions and advanced primary tumors, but its expression was rarely detected in benign lesions (Lehmann et al., 1987). Later studies have demonstrated that CD146 is highly expressed on a variety of carcinomas in addition to melanoma, and therefore CD146 has been suggested as a potential marker for tumor diagnosis, prognosis and treatment (Wang and Yan, 2013). However, recent reports have shown that CD146 not only just acts as a cell adhesion molecule to mediate cell-cell interaction and cell-matrix adhesion, it also acts as a cell surface receptor to transduce extracellular signals (St Croix, 2015; Tu et al., 2015). It is actively involved in many physiological processes such as organ development, cell migration, angiogenesis, immune response and tissue repair (St Croix, 2015; Tu et al., 2015).

In the central nervous system, CD146 has been identified as a recruiter of T cells and it is associated with multiple sclerosis, a central nervous system auto-inflammatory disease (Breuer et al., 2018; Zondler et al., 2020). Elevated soluble CD146 in the cerebrospinal fluid is considered as a biomarker of neuroinflammation and blood-brain barrier dysfunction (Duan et al., 2013; Wang D. et al., 2020). Knockout CD146 in the central nervous system impaired mouse appetite, locomotion and spatial learning abilities (Tu et al., 2013). Expression of CD146 mRNA is increased in the spinal cord following spinal cord injury and it promotes neurogenesis and angiogenesis (Liu et al., 2016). Inhibition of CD146 function down-regulated the expression of angiogenesis-related factors such as VEGFR-2, p-p38 and p-AKT, and the inflammatory factors TNF-a, IL-1b, and IL-8, indicating that the up-regulation of CD146 has a beneficial role in the recovery from spinal cord injury (Liu et al., 2016). These studies revealed an important function of CD146 to promote the repair of central nervous system following injury or nerve degeneration.

Peripheral nerve injury induces vigorous gene expression changes not only in injured neurons but also in cells of the distal nerve stump (Jessen and Mirsky, 2016; Renthal et al., 2020; Toma et al., 2020; Chen et al., 2021). Previously, we studied gene expression changes in both DRG and the distal nerve stump in the rat by microarray analysis, and identified CD146 as one of DEGs in the distal nerve stump (Yi et al., 2015). However, the biological functions of CD146 in peripheral nerve regeneration remain undetermined. In this report, we examined cell type specific up-regulation of CD146 in injured mouse peripheral nervous system. We observed enhanced expression of CD146 in the nerve bridge and in the distal nerve stump after mouse sciatic nerve transection injury. We identified Schwann cells and cells associated with blood vessels expressing high level of CD146 in injured mouse peripheral nerves. An earlier study demonstrated that CD146 knockdown caused cell cycle arrest and induced the growth inhibition of glioma stem cells (Yawata et al., 2019). Similar to the function of CD146 in glioma stem cell proliferation, here, our CCK8 and EdU results showed that silencing of CD146 led to decreased cell viability and proliferation rate of cultured rat primary Schwann cells. Therefore, up-regulated CD146 in Schwann cells after nerve injury may contribute to the enlargement of Schwann cell population to promote peripheral nerve regeneration.

Our bioinformatic and functional pathway analysis revealed that CD146 has an important role in regulating cell migration, and our immunostaining results showed that CD146 is highly expressed on migrating Schwann cells of the nerve bridge, suggesting that CD146 could regulate Schwann cell migration in the nerve bridge following peripheral nerve transection injury. However, both Transwell migration assay and wound healing assay confirmed that knockdown of CD146 in cultured Schwann cells enhanced the speed of their migration, suggesting that CD146 mediated Schwann cell-Schwann cell adhesion could slows down Schwann cell migration in the nerve bridge. Previous studies also reported an opposite effects of CD146 on cell migration, which depends on the cell types of CD146 expression. For instance, it is reported that CD146 motivates the migration of endothelial cells (Zhang et al., 2018) and neuroendocrine carcinoma cells (Piao et al., 2019) but suppresses the migration of pancreatic cancer-associated fibroblasts (Zheng et al., 2016). Schwann cell migration in the nerve bridge is a highly coordinated process. They attach to each other through long bipolar processes and form chains migrating toward the middle of the nerve bridge (Chen et al., 2019, 2020; Min et al., 2020). Cell adhesion molecules play a key role in Schwann cell-Schwann cell interaction and Schwann cell chain migration in the nerve bridge (Parrinello et al., 2010). Thus, despite CD146 mediated Schwann cell adhesion slows down the speed of Schwann cell migration, it could be a key mediator for the formation of Schwann cell chains in the nerve bridge, but this remains to be investigated *in vivo*.

In this study, we show that CD146 is highly up-regulated in Schwann cells and cells associated with blood vessels following peripheral nerve injury. Previously, Nerve growth factor (NGF) has been shown to induce CD146 expression in Schwann cells (Hiroi et al., 2005; Taira et al., 2005). However, any other signals that could directly up-regulate CD146 expression in cells of the distal nerve have not been investigated. Evidence of CD146 expression in other tissue indicates that pro-inflammatory cytokines could also regulate CD146 up-regulation in the distal nerve stump. The production of pro-inflammatory cytokines is induced at the injury site and in the distal nerve stump following peripheral nerve injury (Cheepudomwit et al., 2008; Gaudet et al., 2011; Rotshenker, 2011). Reports have shown that some pro-inflammatory cytokines are able to induce CD146 expression, both tumor necrosis factor-alpha (TNFα) and interleukin-1a (IL1a) could significantly up-regulate CD146 expression in luteinizing granulosa cells (Melnikova et al., 2009; Wang and Yan, 2013). Transforming growth factor-beta (TGFβ) has been shown to up-regulate CD146 expression in hepatocytes (Tsuchiya et al., 2007). IL13 has been reported to induce CD146 expression in airway epithelial cells, this process could promote bacterial adherence to human bronchial epithelial cells (Simon et al., 2011). In addition, reports have shown that endothelin-1 and endothelin-3could enhance CD146 expression in melanocytes (Mangahas et al., 2004; Williams et al., 2014). Thus, growth factors and pro-inflammatory cytokines that are up-regulated in the injured peripheral nerve could regulate CD146 expression in Schwann cells and cells associated with blood vessels.

Taken together, we systematically studied the up-regulation of CD146 in injured mouse peripheral nervous system, and demonstrated that CD146 is up-regulated in Schwann cells and cells associated with blood vessels. We further investigated the biological functions of CD146 on Schwann cell viability, proliferation and migration, and indicate that CD146 could play an essential role in peripheral nerve regeneration.

## Data Availability Statement

The original contributions presented in the study are included in the article/supplementary material, further inquiries can be directed to the corresponding author/s.

## Ethics Statement

The animal study was reviewed and approved by Lab Animal Ethical Committee of Nantong University, and Administration Committee of Experimental Animals, Jiangsu, China. Written informed consent was obtained from the owners for the participation of their animals in this study.

## Author Contributions

XD and JH conceived, analyzed the data, contributed reagents, materials, and analysis tools, wrote the manuscript, and designed the experiments. YS, JZ, QL, SD, and XD performed the experiments. All authors contributed to the article and approved the submitted version.

## Conflict of Interest

The authors declare that the research was conducted in the absence of any commercial or financial relationships that could be construed as a potential conflict of interest.

## Publisher’s Note

All claims expressed in this article are solely those of the authors and do not necessarily represent those of their affiliated organizations, or those of the publisher, the editors and the reviewers. Any product that may be evaluated in this article, or claim that may be made by its manufacturer, is not guaranteed or endorsed by the publisher.

## Footnotes

1
https://satijalab.org/seurat/
